# Genetic modifiers in rare disorders: the case of fragile X syndrome

**DOI:** 10.1038/s41431-020-00711-x

**Published:** 2020-08-29

**Authors:** Hayley Crawford, Gaia Scerif, Lucy Wilde, Andrew Beggs, Joanne Stockton, Pria Sandhu, Lauren Shelley, Chris Oliver, Joseph McCleery

**Affiliations:** 1 Faculty of Health and Life Sciences, Coventry University, Coventry, UK; 2 Warwick Medical School, University of Warwick, Warwick, UK; 3 Cerebra Centre for Neurodevelopmental Disorders, School of Psychology, University of Birmingham, Birmingham, UK; 4 Attention, Brain and Cognitive Development Group, Department of Experimental Psychology, University of Oxford, Oxford, UK; 5 The Open University, Milton Keynes, UK; 6 Surgical Research Laboratory, Institute of Cancer and Genomic Sciences, University of Birmingham, Birmingham, UK; 7 Department of Psychology, Saint Joseph’s University, Philadelphia, PA USA; 8 Center for Autism Research, Children’s Hospital of Philadelphia, Philadelphia, PA USA

**Keywords:** Risk factors, ADHD, Autism spectrum disorders, Genetic markers, Behavioural genetics

## Abstract

Methods employed in genome-wide association studies are not feasible ways to explore genotype–phenotype associations in rare disorders due to limited statistical power. An alternative approach is to examine relationships among specific single nucleotide polymorphisms (SNPs), selected a priori, and behavioural characteristics. Here, we adopt this strategy to examine relationships between three SNPs (5-HTTLPR, MAOA, COMT) and specific clinically-relevant behaviours that are phenotypic of fragile X syndrome (FXS) but vary in severity and frequency across individuals. Sixty-four males with FXS participated in the current study. Data from standardised informant measures of challenging behaviour (defined as physical aggression, property destruction, stereotyped behaviour, and self-injury), autism symptomatology, attention-deficit-hyperactivity-disorder characteristics, repetitive behaviour and mood/interest and pleasure were compared between each SNP genotype. No association was observed between behavioural characteristics and either 5-HTTLPR (serotonin) or MAOA (monoamine oxidase) genotypes. However, compared to the COMT (dopamine) AG and GG genotypes, the AA genotype was associated with greater interest and pleasure in the environment, and with reduced risk for property destruction, stereotyped behaviour and compulsive behaviour. The results suggest that common genetic variation in the COMT genotype affecting dopamine levels in the brain may contribute to the variability of challenging and repetitive behaviours and interest and pleasure in this population. This study identifies a role for additional genetic risk in understanding the neural and genetic mechanisms contributing to phenotypic variability in neurodevelopmental disorders, and highlights the merit of investigating SNPs that are selected a priori on a theoretical basis in rare populations.

## Introduction

With recent advances in genotyping technologies, the study of genotype–phenotype relationships has received significant attention in both the general population and the field of neurodevelopmental and psychiatric disorders. The extant research has employed a variety of methods, including detailed behavioural phenotyping of individuals with single and multiple gene disorders, genome wide association studies (GWAS) and examining phenotypic differences related to individual differences in single nucleotide polymorphism (SNP) genotypes. These lines of research have each identified putative relationships between genetic factors and various aspects of cognitive, social, emotional and behavioural functioning [1–3].

Empirical research delineating behavioural phenotypes has uncovered strong evidence for features that are characteristic of different genetic disorders. For example, syndrome-specific patterns include almost universal levels of self-injurious behaviour in Lesch–Nyhan syndrome, a threefold increase in aggression in individuals with Angelman syndrome, and elevated stereotyped behaviour in Rubinstein–Taybi syndrome [2]. Despite the well-defined behavioural phenotypes associated with genetic syndromes, there is individual within-disorder variability in the presence, severity and frequency of some behaviours. For example, autism symptomatology is heightened in individuals with fragile X syndrome (FXS) and, although traits of autism have been reported in ~75% of individuals with FXS [4], only 30% display these traits at the severity required to meet cut-off for an autism spectrum disorder (ASD) [5]. The factors that protect some individuals and place others with the same monogenic disorder at risk are currently unknown. Identifying additional genetic risk for these and other behavioural characteristics may further understanding of the mechanisms contributing to phenotypic variability within these populations. However, statistical modelling has indicated that GWAS studies should utilise sample sizes between several thousands to more than tens of thousands to effectively answer simple questions, and in the range of 50,000–100,000 or larger to answer more complex questions [6]. The required sample sizes for GWAS would, therefore, be impossible to achieve in studies of individual rare diseases and disorders.

An alternative approach to GWAS in uncovering genotype–phenotype relationships, which offers the opportunity to capture individual variability in behavioural characteristics, is through examining relationships among specific SNPs and behavioural characteristics. Here, SNPs are selected a priori, on a theoretical basis with regard to their functions and previous findings, as evidence suggests many SNPs directly or indirectly affect neurotransmitter function and other neural mechanisms that may play a particular role in phenotypic expression. Our work, and that of others, has identified that normal genotypic variation is associated with differences in important behaviours in typically developing populations [7, 8].

SNP genotype–phenotype research has recently been extended to examine how common variation in SNP genotypes impact behaviour within the context of genetically-mediated syndromes. For example, Hessl et al. [9] reported an association between variation of serotonin-related 5-HTTLPR genotype and aggressive and stereotyped behaviour in FXS. Another study uncovered an association between this SNP genotype and improvements in clinical measures when children with FXS received medication versus a placebo [10]. Studies of SNP genotype–phenotype relationships have also been subject to some degree of non-replication, likely due to variations in study design, different populations and different definitions of phenotypes [11]. In the field of neurodevelopmental disorders, where populations and phenotypic characteristics are often defined at a fine-grained level and samples are less readily available, there have been limited attempts at replication.

### Fragile X syndrome as a model to investigate polymorphism-behaviour relationships

Affecting ~1 in 4000 males and 1 in 8000 females, FXS is the most common cause of inherited intellectual disability [12]. It is caused by an expansion of cytosine–guanine–guanine repeats on the *FMR1* gene. FXS is associated with a number of clinically significant behaviours, including self-injury and aggressive behaviour, ASD symptomatology, hyperactivity and impulsivity, and social anxiety [13]. Despite comparatively heightened prevalence rates in FXS, the nature and severity of these behaviours varies across individuals. The role of secondary genes, namely those associated with the serotonin-transporter-linked polymorphic region (5-HTTLPR) and Monoamine oxidase A (MAOA), has been investigated to further explain the variability of aggressive, self-injurious and stereotypic behaviours in FXS [9]. This previous study reported an association between the L genotype of 5-HTTLPR and aggressive and stereotyped behaviour, but no association between these behaviours and MAOA.

Interestingly, the relationship between 5-HTTLPR and aggressive behaviour reported in this previous study is divergent with that reported in the general population. The present study aims to replicate these findings, and expand on them by exploring the role of 5-HTTLPR and MAOA genotypes for a wider range of behavioural characteristics, which are variably associated with the behavioural phenotype of FXS. This includes challenging behaviour (defined here as physical aggression, property destruction, stereotyped behaviour and self-injury), ASD characteristics, attention-deficit-hyperactivity-disorder (ADHD) characteristics, repetitive behaviour and mood/interest and pleasure. In addition, the present study will delineate the role of the dopamine-related SNP, COMTVal158Met, in these behavioural characteristics, which has not yet been explored in FXS but has been associated with behaviour in other populations. See Supplementary information File 1 for additional information on 5-HTTLPR, MAOA and COMT including background and existing literature. Relationships between each of these three SNPs in the expression of clinically relevant behaviours, including those measured here, are reported in different populations. However, most of these relationships have been not been replicated, likely due to differences in definitions of phenotypes and measures used to capture phenotypic data, as well as insufficiently powered studies, and variability in study samples. Interestingly, the possibility of the same candidate gene or DNA variant being associated with different risks in different populations has been highlighted [11].

To our knowledge, only one study has investigated the role of variation in SNP genotypes to phenotypic variation in FXS [9]. Based on this, it was hypothesised that the L genotype of 5-HTTLPR will be associated with aggressive and stereotyped behaviour whilst no association between challenging behaviour and MAOA will be found. The role of 5-HTTLPR and MAOA in other behavioural characteristics (autism symptomatology, ADHD characteristics, repetitive behaviour and mood disorders) has not been investigated in FXS. Therefore, secondary hypotheses for these additional behaviours were based on research conducted in other populations. Such research indicates that: (a) the 5-HTTLPR S allele will be associated with ADHD characteristics and mood/interest and pleasure, whereas the L allele will be associated with repetitive behaviour, (b) the 4-repeat MAOA allele will be associated with ASD, (c) the AA COMT genotype will be associated with challenging behaviour, ADHD characteristics and mood/interest and pleasure whereas the GG genotype would be associated with repetitive behaviour. No hypotheses were generated for additional polymorphism–behaviour relationships due to a lack of literature and/or mixed results.

## Methods

### Participants

This study was conducted as part of a large-scale questionnaire study investigating behaviour in individuals with a range of different neurodevelopmental disorders. Parents and carers of males with FXS were recruited to the questionnaire study through the Fragile X Society, the UK family support group. Overall, parents or carers of 252 males with FXS returned an eligible questionnaire pack. For the current study, each of these families were contacted directly and asked if they would be willing to provide a saliva sample from their child or the person they care for. One hundred and two families agreed to take part in this phase of the project. Telephone contact was attempted with all 102 families that initially agreed to take part. Fourteen families were unable to collect saliva due to behavioural challenges. Saliva samples were returned from 64 males with FXS (62.75%). The remaining families were either not contactable or they verbally agreed to return saliva samples but these were not received. A diagnosis of FXS was confirmed through genetic testing for all participants. The demographic characteristics of the participants are displayed in Table 1.

**Table 1:** Participant characteristics for the full sample.

Participant characteristic (*n* = 64)	
Chronological age mean (SD)^a^	18.11 (9.67)
Range	3.93–41.24
% Verbal (speak/sign more than 30 words)^a^	89.1
% Mobile (walk unaided)^a^	96.9
% Partly able/able^b^	89.1
% Meeting cut off for ASD^c^	70.3
% Meeting cut off for autism^c^	31.3
% displaying self-injurious behaviour within last month^d^	43.8
% displaying physical aggression within last month^d,e^	40.6
% displaying destruction of property within last month^d,f^	34.4
% displaying stereotyped behaviour within last month^d^	60.9

^a^Chronological age, verbal and mobility data obtained from the Demographic Questionnaire. Age data missing from one participant.

^b^Ability measured by the self-help subscale of the Wessex Scale.

^c^ASD and autism cut off data obtained from the Social Communication Questionnaire (SCQ). SCQ data are not available for six participants.

^d^Data on challenging behaviour including self-injurious behaviour, physical aggression, destruction of property and stereotyped behaviour obtained from the Challenging Behaviour Questionnaire.

^e^Physical aggression data not available for two participants.

^f^Destruction of property data not available for one participant.

### Measures

Each participant’s primary caregiver completed a demographic questionnaire, the Wessex Scale, the Challenging Behaviour Questionnaire (CBQ), the Social Communication Questionnaire (SCQ), the Activity Questionnaire (TAQ), the Repetitive Behaviour Questionnaire (RBQ) and the Mood, Interest and Pleasure Questionnaire-Short Form (MIPQ-S). See Supplementary information File 2 for additional information on the questionnaire measures.

### Procedure

Parents and carers of males with FXS that agreed to take part in the study were sent an Oragene DNA (OG-575) saliva self-collection kit with accompanying instructions on using the device and packaging the sample for postal return. Families that provided saliva samples were contacted to request retrospective information on the participant’s medication use.

### Genetic analysis

Saliva samples were analysed to confirm a diagnosis of FXS, as well as to identify the 5-HTTLPR (S/S, S/L, L/L), MAOA (number of repeats) and COMT (AA, AG, GG) genotypes. DNA was extracted from saliva samples using PrepIT L2 protocol from DNA Genotek ON, Canada according to the manufacturer’s instructions. Fragile X analysis was performed using primers described in Hantash et al. [14]. The PCR was performed using Fast Start polymerase (Roche) under the following conditions: 2× Buffer, 90 mM MgCl, 20% Q-solution (Qiagen), 1% Deaza dGTP (NEB), 10% DMSO, 25 mM each primer, 1U Taq with 20 ng DNA. Cycling conditions were as follows (with the ramp speed adjusted to 2°/s) 95 °C 15 min, followed by 2 cycles of 95 °C 30 s, 65 °C 30 s 72 °C, then a 2° decrease in annealing temp every 2 cycles plus an increase in cycle number for the next eight rounds, followed by a final extension of 10 min 72 °C. Analysis of the fragments was performed using an ABI 3730 sequencer and analysed using Genemapper software 5 and Rox500 size standard (Applied Biosystems).

COMT analysis was performed using TaqMan genotyping (assay ID C__25746809_50) according to the manufacturer’s instructions. 5HTTLPR analysis was performed as described in Wray et al. [15]. MAOA analysis was performed using primers described in Guo et al. [16]. The PCR was performed using Fast Start polymerase (Roche) under the following conditions: 2x Buffer, 90 mM MgCl, 20% Q-solution (Qiagen), 1% Deaza dGTP (NEB), 10% DMSO, 25 mM each primer, 1U Taq with 20 ng DNA. Cycling conditions were as follows: 95 °C 10 min, followed by 2 cycles of 95 °C 30 s, 65 °C 30 s 72 °C 1 min, then a 2° decrease in annealing temp every 2 cycles plus an increase in cycle number for the next seven rounds, with a final amplification on 25 cycles of 95 °C 30 s, 56 °C 30 s and 72 °C 1 min. Analysis of the fragments was performed using an ABI 3730 sequencer and analysed using Genemapper software 5 software and Rox500 size standard (Applied Biosystems).

### Data analysis

There were five sibling pairs in the participant sample (four twin pairs). For reporting the prevalence of different genotypes in the same, one sibling from each pair was randomly removed to avoid reporting bias. However, data from all 64 participants were included in the genotype-phenotype analyses based on the assumption that the behavioural phenotype and, therefore, the genotype-phenotype relationship is unlikely to be identical between siblings.

The distribution of questionnaire data was inspected for normality via the One-Sample Kolmogorov–Smirnov test. Data from the following subscales and total scores were normally distributed (*p* > 0.05): TAQ Total Score, MIPQ-S Interest and Pleasure subscale, RBQ Repetitive Language subscale, RBQ Total Score, SCQ Total Score. Data from all other subscales and total scores were not normally distributed (*p* < 0.05).

For genotype-phenotype analyses, one-way ANOVAs and Kruskal-Wallis tests were conducted to assess differences between the different 5-HTTLPR and COMT genotypes at full scale and subscale level of each questionnaire measure that yields continuous data (CBQ self-injury severity, SCQ, TAQ, RBQ, MIPQ). Where differences existed, independent samples *t*-tests and Mann–Whitney tests were conducted to locate the source of difference. The majority of participants expressed either 3 or 4 MAOA repeats. Independent samples *t*-tests and Mann–Whitney tests were conducted to assess differences between those with three repeats and those with four repeats at full scale and subscale level of each questionnaire measure yielding continuous data. Chi-square tests were used to analyse differences between 5-HTTLPR, COMT, and MAOA genotypes and categorical items on the CBQ (presence of self-injury, destruction of property, physical aggression and stereotyped behaviour).

The sample size reported here is the largest for studies of SNPs in FXS, and is similar to or larger than the sample size for existing published studies of other neurodevelopmental disorders including autism (*n* = 73 [17]; *n* = 41 [18]). A post-hoc power analysis was performed for power estimation given the available sample size. Assuming a medium effect size of 0.5, and a *p* value of 0.05, power was determined to be 0.95 for ANOVAs with three participant groups (5-HTTLPR and COMT analyses) and 0.41 for two groups (MAOA analysis).

## Results

Kruskal–Wallis and Mann–Whitney tests revealed that neither chronological age nor ability level, as measured by the Wessex, was associated with either the 5-HTTLPR, MAOA or COMT genotypes (all *p* > 0.05). Table 2 presents the mean subscale and full-scale scores for each measure as a function of 5-HTTLPR, MAOA and COMT genotypes.

**Table 2:** Mean scores and standard deviations (in parentheses) for each behavioural measure at subscale level as a function of genotype.

	5-HTTLPR			MAOA		COMT		
	L/L (*n* = 21)	L/S (*n* = 24)	S/S (*n* = 19)	3 (*n* = 21)	4 (*n* = 39)	AA (*n* = 11)	AG (*n* = 41)	GG (*n* = 11)
Chronological age (years)	18.25 (10.04)	16.13 (8.62)	18.84 (11.05)	16.81 (8.96)	18.50 (10.42)	16.55 (6.56)	18.02 (10.06)	18.60 (11.48)
The Wessex Scale								
Self-help	7.57 (1.29)	7.04 (1.33)	7.58 (1.50)	6.95 (1.50)	7.59 (1.31)	7.18 (1.60)	7.41 (1.30)	7.45 (1.57)
The Activity Questionnaire								
Impulsivity	15.38 (7.72)	17.54 (5.20)	13.11 (7.65)	17.00 (6.12)	14.59 (7.44)	16.36 (6.95)	16.49 (6.38)	11.82 (8.34)
Overactivity	15.58 (9.73)	17.50 (10.31)	16.13 (9.47)	17.24 (9.72)	15.97 (9.95)	16.27 (10.71)	17.21 (9.78)	14.82 (9.28)
Impulsive Speech^a^	4.37 (3.06)	5.95 (3.99)	3.69 (2.60)	4.37 (2.81)	4.79 (3.65)	3.91 (3.18)	5.58 (3.57)	3.22 (2.05)
Total Score	34.92 (18.11)	40.50 (16.59)	32.34 (16.64)	38.19 (14.00)	34.74 (18.57)	36.55 (18.12)	38.60 (16.65)	29.27 (17.21)
Mood, Interest and Pleasure Questionnaire								
Mood	20.29 (2.67)	19.96 (3.68)	20.54 (2.24)	20.14 (3.57)	20.34 (2.68)	21.09 (2.02)	19.88 (3.25)	20.38 (2.37)
Interest and Pleasure	16.86 (3.73)	14.87 (3.74)	17.13 (3.88)	16.52 (4.14)	16.06 (3.84)	18.91* (3.08)	15.28* (3.93)	16.55 (3.08)
Total Score	37.14 (5.28)	34.83 (6.72)	37.67 (4.98)	36.67 (7.21)	36.40 (5.32)	40.00* (4.31)	35.15* (6.03)	36.93 (4.87)
Repetitive Behaviour Questionnaire								
Stereotyped Behaviour	5.76 (2.96)	6.96 (4.08)	5.94 (4.37)	6.52 (3.76)	6.33 (3.76)	3.73* (3.64)	6.58* (3.73)	8.22* (3.07)
Compulsive Behaviour	6.29 (8.60)	7.00 (6.41)	6.03 (6.71)	7.00 (7.49)	6.72 (7.30)	2.45* (4.82)	7.64* (7.85)	6.70* (5.46)
Insistence on Sameness	4.43 (2.62)	4.42 (2.95)	4.00 (2.93)	4.38 (2.44)	4.34 (3.02)	3.18 (2.14)	4.85 (3.00)	3.70 (1.89)
Restricted Preferences^a^	4.89 (4.33)	5.95 (4.20)	3.63 (3.43)	5.74 (3.71)	4.83 (4.28)	4.68 (3.41)	4.97 (4.32)	5.88 (4.12)
Repetitive use of Language^a^	5.87 (3.76)	7.48 (3.31)	5.87 (4.07)	7.47 (4.14)	5.92 (3.30)	6.82 (3.40)	6.84 (3.73)	4.88 (4.02)
Total Score	26.60 (16.84)	30.83 (17.50)	24.07 (17.31)	30.24 (16.91)	27.04 (17.38)	20.86 (14.49)	29.85 (18.24)	27.78 (14.08)
Social Communication Questionnaire								
Communication	6.55 (2.39)	7.19 (2.01)	5.64 (3.31)	7.32 (2.27)	6.24 (2.58)	6.26 (1.39)	6.93 (2.72)	5.85 (2.46)
Restricted, Repetitive and Stereotyped Behaviours	3.90 (2.23)	4.71 (2.07)	4.53 (3.36)	4.14 (2.22)	4.59 (2.68)	3.73 (2.37)	4.71 (2.61)	4.09 (2.55)
Social Interaction	7.82 (3.19)	8.36 (2.56)	6.73 (3.99)	8.29 (2.70)	7.44 (3.34)	7.27 (2.24)	8.29 (3.38)	6.56 (3.13)
Total Score	18.80 (6.61)	20.97 (5.19)	17.68 (9.69)	20.55 (5.17)	18.84 (7.50)	18.26 (3.61)	20.76 (7.52)	15.83 (6.37)
Challenging Behaviour Questionnaire								
Self-Injury Severity Score^b^	4.95 (2.10)	5.42 (2.19)	6.14 (2.10)	5.65 (2.16)	5.35 (2.26)	4.79 (1.89)	5.64 (2.33)	5.63 (1.60)
% Self-Injury^c^	47.62	50.00	31.58	47.62	41.03	54.55	43.90	36.36
% Physical Aggression^c^	45.00	33.33	50.00	38.10	40.54	40.00	43.90	40.00
% Destruction of Property^c^	38.10	37.50	27.78	23.81	42.11	0.00*	46.34*	30.00*
% Stereotyped Behaviour^c^	66.67	58.33	57.89	57.14	64.10	27.27*	68.29*	72.72*

NB Data missing from between none and six participants, depending on subscale.

*p < 0.05.

^a^Scored for verbal participants only.

^b^Self-injury severity score is only calculated for participants displaying self-injury within the last month.

^c^Percentage of participants expressing given genotype showing this behaviour in the last month.

### 5-HTTLPR

Excluding one from each sibling pair (total *n* = 59), 5-HTTLPR genotyping revealed 18 participants homozygous for the L/L genotype (30.51%), 18 participants homozygous for the S/S genotype (30.51%) and 23 participants with the L/S genotype (38.98%).

Including all participants (*n* = 64), no significant differences were revealed between the three genotype groups on the questionnaire measures at subscale level (all *p* > 0.05). In addition, no significant differences were revealed on categorical items of the CBQ assessing the presence of self-injury, destruction of property, aggression and stereotyped behaviour (all *p* > 0.05). Power estimation for this analysis was high (0.95) and significant effects of 5-HTTLPR and challenging behaviour have previously been reported in a smaller sample size of 47 males with FXS [9]. Therefore, statistical power is not thought to underlie the null results.

### MAOA

Excluding one from each sibling pair (total *n* = 59), MAOA genotyping revealed 19 participants with 3 repeats (32.20%), 37 participants with 4 repeats (62.71%), two participants had 3.5 repeats (3.39%), and one participant had 5 repeats (1.69%). Due to the small numbers of participants expressing 3.5 and 5 MAOA repeats, the genotype–phenotype analysis included comparisons only between those with three and those with four repeats.

Including all eligible data (*n* = 61), no significant differences were revealed between these two genotype groups on the questionnaire measures at subscale level (all *p* > 0.05). In addition, no significant differences were revealed on the categorical items assessing the presence of any form of challenging behaviour (all *p* > 0.05). Power estimation for these analyses was low and so caution should be applied when interpreting these results.

### COMT

COMT genotype information could not be obtained for one participant. Excluding one from each sibling pair (total *n* = 58), COMT genotyping revealed nine participants homozygous for the A/A genotype (15.52%), 11 homozygous for the G/G genotype (18.97%) and 38 with the A/G genotype (65.52%).

**Fig. 1:**
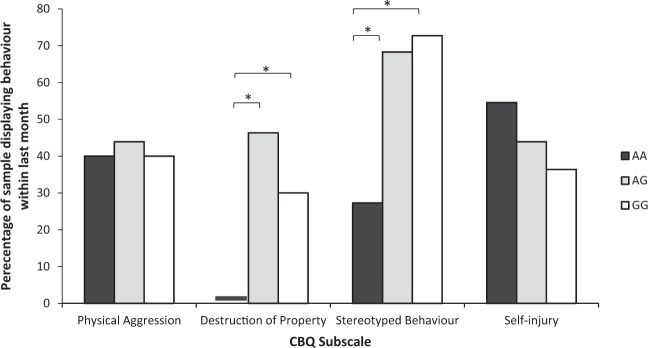
Mean scores on each subscale of the Challenging Behaviour Questionnaire for each COMT genotype group. The mean score for Destruction of Property in the AA genotype group was zero.

Chi-square tests including all participants (*n* = 63), revealed a significant difference between the COMT genotypes on the presence of destruction of property (*χ*^2^(2) = 8.293, *p* = 0.016) and stereotyped behaviours measured by the CBQ (*χ*^2^(2) = 6.850, *p* = 0.033; Fig. 1). Follow-up analyses revealed that this significant difference was driven by lower scores, and therefore *lower* levels of property destruction and stereotyped behaviour, in those with the AA versus both the AG genotype (destruction of property: *χ*^2^(1) = 8.033, *p* = 0.005; stereotyped behaviour: *χ*^2^(1) = 6.062, *p* = 0.014) and the GG genotype, although this difference was marginal for destruction of property (destruction of property: *χ*^2^(1) = 3.850, *p* = 0.050; stereotyped behaviour: (*χ*^2^(1) = 4.545, *p* = 0.033).

**Fig. 2:**
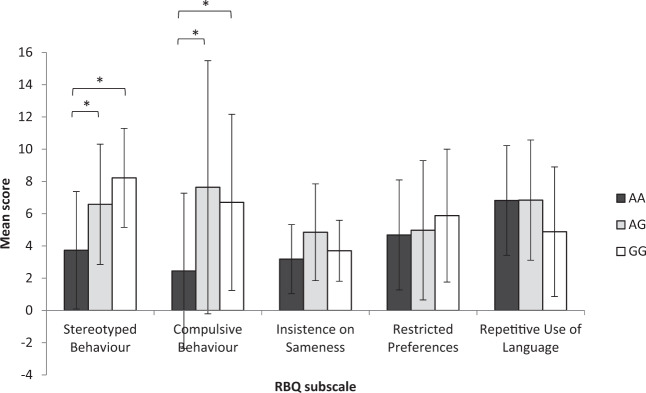
Mean scores on each subscale of the Repetitive Behaviour Questionnaire for each COMT genotype group. Error bars represent standard deviation.

Significant differences were also revealed between the COMT genotypes and Stereotyped Behaviour (*χ*^2^(2) = 7.790, *p* = 0.020) and Compulsive Behaviour (*χ*^2^(2) = 6.927, *p* = 0.031) subscales of the RBQ (Fig. 2). Individuals with the AA genotype scored significantly lower than those with the AG and GG phenotypes on both the Stereotyped Behaviour subscale (AA vs. AG: *U* = 126.500, *p* = 0.031; AA vs. GG: *U* = 14.500, *p* = 0.006) and the Compulsive Behaviour subscale (AA vs. AG: *U* = 115.000, *p* = 0.014; AA vs. GG: *U* = 22.000, *p* = 0.020). These results indicate that those with the AA genotype display *lower* levels of stereotyped and compulsive behaviour than those with the AG or GG genotypes.

**Fig. 3:**
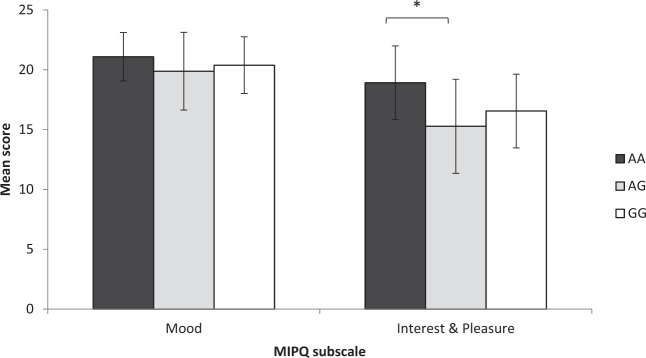
Mean scores on each subscale of the Mood, Interest and Pleasure Questionnaire for each COMT genotype group. Error bars represent standard deviation.

Significant differences were also revealed between COMT genotypes and the Interest and Pleasure subscale (*F* (2, 62) = 4.338, *p* = 0.017) of the MIPQ-S (Fig. 3). Specifically, those with the AA genotype scored significantly higher, indicating *higher* interest and pleasure, than those with the AG phenotype on the Interest and Pleasure subscale (*p* = 0.015).

### Medication

As medication may impact on the behaviours being assessed in the current study, retrospective medication data were obtained from 60 participants. All analyses were re-conducted excluding data from 11 participants that took psychoactive medication within six months of questionnaire completion. The pattern of results remained largely unchanged. The only distinctions were (1) differences between COMT genotypes and the stereotyped behaviour subscale of the CBQ were no longer significant (*p* > 0.05), (2) lower levels of insistence of sameness in individuals with the AA COMT genotype compared to those with the AG genotype (*χ*^2^(2) = 6.302, *p* = 0.043; AG versus AA: *U* = 61.000, *p* = 0.038), and (3) differences between COMT genotypes and interest and pleasure were no longer significant (*p* > 0.05).

## Discussion

Here, we present examinations of putative genotype–phenotype associations between three SNPs (5-HTTLPR, MAOA and COMT), selected a priori, and a range of clinically relevant behaviours in males with FXS to identify additional genetic risk for these behavioural characteristics. Males with FXS are at high risk of displaying each of the behavioural characteristics investigated in the current study, namely, challenging behaviour (defined here as physical aggression, property destruction, stereotyped behaviour, and self-injury), autism symptomatology, ADHD characteristics, repetitive behaviour and low mood. However, the frequency and severity of each of these behavioural characteristics is variable within the FXS population. Our results indicate that common genetic variation in the dopamine-related COMT genotype contributes meaningfully to clinical variability in challenging and repetitive behaviours and interest and pleasure characteristics in this population. Specifically, compared to the AG and GG genotypes, the AA genotype was associated with reduced risk for property destruction, stereotyped and compulsive behaviour, and with greater interest and pleasure in the environment. No association was observed between behavioural characteristics and either serotonin-related (5-HTTLPR) or monoamine oxidase related (MAOA) genotypes. The results of the current study contribute to the emerging field on personalised treatments and, if replicated, highlight that variation in COMT may inform tailored interventions. Understanding the genetic basis for variable behavioural expression across people with FXS has implications for early detection of the heightened risk for these and other clinically significant behaviours, which can ultimately optimise outcomes for these individuals through biologically informed and preventative interventions.

### 5-HTTLPR

The lack of association between the 5-HTTLPR genotype and challenging behaviour is inconsistent with the one existing study investigating this relationship in males with FXS [9]. This previous study reported that individuals with the L/L genotype displayed significantly higher levels of aggressive and destructive behaviour than those with the S/S genotype, and higher levels of stereotypic behaviour than those with the S/L genotype. A potential reason for this discrepancy is differences in population characteristics. Specifically, 75 and 98% of the sample reported in Hessl et al. [9] demonstrated aggressive and stereotyped behaviour, respectively, within a two-month period. In contrast, only 40 and 60% of the sample included in the current study demonstrated aggressive and stereotyped behaviour, respectively, within the previous month. Thus, the overall severity of challenging behaviour was substantially higher in the study sample reported by Hessl et al. [9] than in the current sample. In addition, the measures used to capture information on property destruction, self-injurious and aggressive behaviour differed between the two studies. The study by Hessl et al. [9] used a five-point frequency scale and a four-point severity scale to assess self-injurious behaviour, stereotyped behaviour, and aggression/destruction over the past two months. The current study focussed on the presence of aggressive behaviour and property destruction over the past one month, and the presence and severity of self-injurious behaviour with the latter capturing length of episode, restraint, and frequency on a five-point scale. In the present study, stereotyped behaviour was captured using two instruments, one assessing presence and another assessing frequency on a five-point scale. All these measurement differences may have also contributed to the discrepant findings.

The association between the L/L genotype and aggressive behaviour has also been reported in individuals with ASD [17], but only when using a single parent-interview item and not when this same behaviour was measured via observation. The same relationship has also been reported in individuals with intellectual disability who display aggressive behaviour [19]. Again, the severity of aggressive behaviour may be higher in individuals living in residential homes than with their parents or primary caregivers, as was the case with most participants in the current study. Therefore, one possible explanation for the discrepancy between the  findings reported in the current study and those of Hessl et al. is an interaction among FXS, 5-HTTLPR genotypes and environments, which increase overall rates of challenging behaviour in individuals with FXS. Future studies should, therefore, include measures of participant and environmental factors related to risks for challenging behaviour. In addition, challenging behaviour encapsulates a broad range of behaviours and, therefore, specificity of the construct being measured is important in future studies of genotype–phenotype associations [see 20].

The results reported here do not support existing research conducted in the other populations indicating a relationship between 5-HTTLPR and other behavioural characteristics including ADHD [21–23], ASD symptom severity [17] and depression [24, 25]. Many of these studies were conducted in the general population, which highlights the importance of conducting genotype–phenotype studies in unique, well-defined populations, such as FXS.

### MAOA

The current study revealed no significant differences in behavioural characteristics between individuals with three versus four MAOA repeats. This is consistent with previous findings by Hessl et al. [9]. However, limited statistical power may account for the results in both the existing and current study. Research has generally reported mixed results for MAOA genotypes and aggressive behaviour within the general population, particularly when a direct association between genotype and phenotype is investigated, as effects of MAOA appear more likely to be mediated by environmental interactions [26, 27]. Contradictory results have also been reported in neurodevelopmental disorders with one study reporting a twofold higher risk of autism in individuals with four versus three MAOA repeats [28], and another study reporting that the three repeat allele was associated with increased severity of autism [18]. Individuals with FXS show an atypical profile of ASD-related impairments, which has previously accounted for subtle differences in FXS and idiopathic ASD populations [29], and may explain the lack of association reported here.

### COMT

The results reported here revealed that the AA genotype of COMT is associated with greater interest and pleasure, and with reduced risk for property destruction, compulsive behaviour and stereotyped behaviour. To our knowledge, this is the first study to explore the relationship between genotypes of this dopamine-related SNP and behavioural characteristics in individuals with FXS, and indicates that variation in genetically-mediated dopamine levels in the brain may go some way toward explaining the variability in the presence and severity of a number of behavioural characteristics. Interestingly, the AA genotype has been associated with increased depression in the general population [30, 31], suggesting that the mechanisms contributing to this genotype–phenotype relationship may be different in those with and without FXS. Mixed results have been reported in the general population with regard to other behavioural characteristics and, therefore, the current study expands on this by highlighting a relationship between the AA genotype and compulsive and stereotyped behaviour in individuals with a well-defined genetic syndrome. The AA genotype results in higher dopamine in the prefrontal cortex. Importantly, the effect of these increases in dopaminergic function may be dependent on the level of pre-existing dopamine in the system [32]. For example, increased levels of dopamine, or larger doses of dopamine administered as treatment in clinical populations such as Parkinson’s Disease, have been linked to impulsivity and compulsive behaviour [33, 34], whereas they decrease impulsivity in individuals with ADHD [35]. These findings point to a likely important relationship between dopaminergic function and compulsive behaviour, in which COMT genotypes play a role. However, the exact mechanisms linking COMT, dopaminergic function and compulsive behaviour for individuals with neurodevelopmental disorders requires further investigation.

There are several strengths to this investigation into the role of three distinct SNPs on specific clinically relevant behavioural characteristics, including the a priori selection of SNPs based on existing theoretical bases. In addition, the current study includes the largest FXS sample to date to examine SNPs as a genetic basis for variability in the behavioural phenotype. Finally, the current study used a number of standardised measures, which have been designed specifically for people with intellectual disability, to assess a wide range of behavioural characteristics, covering challenging behaviour, autism symptomatology, ADHD characteristics, repetitive behaviour, and mood/interest and pleasure. A limitation of the current study is the lack of IQ measures to characterise the ability level of participants. However, this was a product of conducting a postal survey in order to maximise the response rate for the largest possible sample size, and the Wessex served as a proxy for intellectual and adaptive functioning. In addition, a number of statistical comparisons were employed to fully investigate the role of 5-HTTLPR, MAOA and COMT variation in behavioural characteristics. The number of comparisons, as well as the modest sample size, particularly in the COMT A/A group, increases the chance of Type 1 error. However, such issues are inevitable when collecting data from individuals with rare genetic syndromes. Applying stringent statistical methods to counteract this may eliminate the reporting of a true association. Here, measures were not taken to account for multiple comparisons. Rather, it is suggested that the results are interpreted with caution and replication is encouraged. In addition, given the challenges associated with collecting saliva samples from individuals with FXS, especially those displaying self-injurious or aggressive behaviour, the sample of this study, as well as other studies utilising similar methodologies, may be biased in such a way that excludes those with the most severe presentations of these behaviours.

## Conclusions

This is the first investigation of the differential association between three SNPs, which affect brain functions, 5-HTTLPR (serotonin), MAOA (monoamine oxidase) and COMT (dopamine), and a wide range of clinically important behavioural characteristics in individuals with FXS, a well-defined genetically mediated syndrome associated with a heightened risk of displaying such behaviours. This study was adequately powered to assess the genotype–phenotype associations and has highlighted a role for the dopamine-related COMT gene, with the AA genotype involved in mediating compulsive, stereotyped and challenging behaviours, as well as interest and pleasure. This highlights the merit of investigating SNPs that are selected a priori on a theoretical basis, in rare populations. Future studies should strive to replicate this finding and explore the association between other SNPs and behavioural phenotypes of FXS and other neurodevelopmental disorders. In addition, the present study failed to replicate previous findings reporting an association between 5-HTTLPR genotypes and challenging behaviour, possibly due to differences in severity of challenging behaviours in the two samples. Future studies should explore this further by examining additional risks for increased severity of challenging behaviour. This study identifies a role for additional neural and genetic risk factors in understanding of the mechanisms contributing to clinically significant phenotypic variability in neurodevelopmental disorders. This increased understanding has implications for timely detection of clinically significant behaviours, which can ultimately improve outcomes through preventative interventions that are biologically informed.

## Supplementary information

Supplementary Information File 1

Supplementary Information File 2
