# Supplementary material for: Genetic modifiers in rare disorders: the case of fragile X syndrome
Source: Eur J Hum Genet. 2020 Aug 29;29(1):173–83. doi: 10.1038/s41431-020-00711-x (PMC7852869; doi:10.1038/s41431-020-00711-x)
Supplement: Supplementary file 1 — Supplementary Information File 1 [file 41431_2020_711_MOESM1_ESM.pdf]

Genetic modifiers in rare disorders: The case of fragile X syndrome

H. Crawford, G. Scerif, L. Wilde, A. Beggs, J. Stockton, P. Sandhu, L. Shelley, C. Oliver & J. P. McCleery

Supplementary Information File 1 Additional information on 5-HTTLPR, MAOA, and COMT: Background and existing literature

5-HTTLPR

5-HTTLPR is a polymorphism in the promotor region of the SLC6A4 gene. This gene codes for the serotonin transporter protein (5-HTT), which facilitates serotonin reuptake from the synapse. Although additional allele subtypes have been identified, two alleles of 5-HTTLPR are commonly investigated in the extant literature: a short (S) variant and a long (L) variant. The S allele is associated with lower transcription and transporter levels, resulting in less serotonin reuptake, whilst the L allele is associated with increased reuptake.[1] Due to the established role of serotonin in the regulation of behaviours related to affective disorders, the role of 5-HTTLPR in normal variation in both social and emotional functioning in the typical population and in neuropsychiatric disorders has been studied fairly extensively with the majority of reports indicating an increased susceptibility for negative social and/or emotional outcomes in individuals with at least one copy of the S-allele.[2–4 but see also 5,6]

Studies investigating the association between 5-HTTLPR and behaviour have indicated that the S-allele is more commonly related to aggressive behaviour than the L-allele in the general population.[7,8] However, studies of FXS and other neurodevelopmental disorders have reported a divergent relationship. Specifically, Hessl et al[9] reported a relationship between the L genotype and aggressive and stereotyped behaviour in individuals with FXS. The L genotype has also been associated with more severe aggressive behaviour in individuals with ASD,[10] and more frequent aggressive behaviour in individuals with intellectual disability (ID).[11] This reversed genotype-phenotype relationship relative to typical individuals may be related to the differing genetic and/or environmental landscapes associated with these neurodevelopmental disorders. For example, both FXS and ASD are associated with elevated levels and unique profiles of anxiety, with other genetic cause, which may affect phenotypic expression of the 5HTTLPR genotype. However, this finding of reverse genotype-phenotype relationships in FXS, ASD, and/or ID requires replication.

The association between variation in 5-HTTLPR and behaviour in FXS has only been explored in relation to aggressive and stereotyped behaviour. However, other behaviours associated with FXS have been investigated in both the general population and in individuals with other neurodevelopmental disorders. This literature reports a relationship between ADHD characteristics and the S genotype when comparing individuals with ADHD and healthy controls,[12,13] and when measuring ADHD symptoms in individuals with ASD.[14] The L/L genotype has been associated with repetitive behaviour in ASD, and the S/S, S/L, and L/L genotypes have each been associated with different aspects of social communication impairment in ASD.[10] The S allele has also been associated with depression[2,15-17] and anxiety[18] in the general population.

MAOA

MAOA is an enzyme that is encoded by the MAOA gene on the X chromosome (Xp11.23), which breaks down neurotransmitters in the monoamine system, including serotonin, dopamine, adrenalin, and noradrenaline, each of which play a role in regulating key behaviours. MAOA has been associated with aggressive behaviour following studies of

MAOA knockout mice demonstrating heightened aggressive behaviour in mice with MAOA knockouts compared to those with MAOB knockouts and their normal counterparts.[19] Although human MAOA knockout is rare, common polymorphisms have been identified. The genetic variation in MAOA consists of a 30 base-pair variable number tandem repeat (VNTR) located in the promotor region of the MAOA gene that can include 2, 3, 3.5, 4, or 5 copies, with the 3 or 4 repeats most commonly found in Caucasians. The presence of 3.5 or 4 repeats is associated with higher expression, whereas 3 repeats results in lower expression.[20] Direct associations between MAOA and aggressive behaviour in humans have yielded mixed results, however gene-environment interactions have revealed an association between low MAOA expression and anti-social behaviour in males experiencing early-life adversity[21] and between low MAOA expression and aggressive behaviour particularly in those who experienced provocation.[22]

Studies investigating the role of MAOA in behavioural variation in neurodevelopmental disorders have reported no association of genotype to aggressive, stereotyped, or self-injurious behaviours in FXS.[9] In ASD, contradictory findings have been reported. Specifically, one study reported an association between the high activity 4-repeat allele and a two-fold increase of risk for autism compared to the low activity 3-repeat allele[23]. Another study reported that males with ASD who had the low activity 3-repeat allele demonstrated more severe sensory behaviour, arousal regulation problems, aggression, and poorer social communication skills than males with the high activity 4-repeat allele.[24] Existing literature has suggested that there is no relationship between MAOA and depression.[25–27]

COMT

Although not examined in FXS, another polymorphism that has gained significant attention in other populations is the Catechol-o-methyl transferase (COMT) Val158Met single nucleotide polymorphism (SNP; rs4680). The COMT gene encodes the COMT enzyme, which breaks down catecholamines, including dopamine, adrenaline, and noradrenaline in the prefrontal regions of the brain. The allelic variation in the Val158Met polymorphism consists of the A (Met) allele and the G (Val) allele. Compared to the Val allele, the Met allele reduces the COMT enzyme activity, which in turn results in lower dopamine-degrading activity and lower prefrontal dopamine receptor density, ultimately resulting in higher dopamine in the prefrontal cortex. COMT genotypes are expressed as either homozygous (AA [MetMet], GG [ValVal]) or heterozygous (ValMet).

Variation in COMT has been associated with a range of behavioural characteristics. Specifically, ADHD characteristics have been associated with both the Val[28] and Met.[29–31] These contradictory results may be partially explained by gender differences as ADHD characteristics have been associated with the Met allele in males,[32] but the Val allele in females.[33] Aggressive behaviour has been associated with the Met genotype in individuals with schizophrenia.[34–36] The role of the dopamine pathway has been particularly implicated in stereotyped behaviour. For example, fewer dopaminergic nerve terminals and cell bodies have been reported in individuals with Lesch-Nyhan syndrome,[37] and this has been proposed to explain the repetitive self-injurious behaviour exhibited in this population. The Val genotype has been associated with stereotyped behaviour,[38] and links between the Met allele and obsessive compulsive disorder in males[39] may point to a link between COMT variation and compulsive behaviour. Contradictory findings have been reported with regard to autism symptomatology with both overrepresentation of Val[40] and Met[31] identified in individuals with ASD. The Met genotype has been associated with depression[41,42] and suicidality.[43]

References

1 Lesch K-P, Bengel D, Heils A, et al. Association of anxiety-related traits with a polymorphism in the serotonin transporter gene regulatory region. *Science* 1996; **274:** 1527–1531.

2 Pezawas L, Meyer-Lindenberg A, Drabant EM, et al. 5-HTTLPR polymorphism impacts human cingulate-amygdala interactions: a genetic susceptibility mechanism for depression. *Nat Neurosci* 2005; **8:** 828.

3 Drabant EM, Ramel W, Edge MD, et al. Neural mechanisms underlying 5-HTTLPR-related sensitivity to acute stress. *Am J Psychiatry* 2012; **169:** 397–405.

4 Clarke H, Flint J, Attwood A, Munafo M. Association of the 5-HTTLPR genotype and unipolar depression: a meta-analysis. *Psychol Med* 2010; **40:** 1767–1778.

5 Munafò MR, Freimer NB, Ng W, et al. 5-HTTLPR genotype and anxiety-related personality traits: A meta-analysis and new data. *Am J Med Genet B: Neuropsychiatr Genet* 2009; **150:** 271–281.

6 Culverhouse RC, Saccone NL, Horton AC, et al. Collaborative meta-analysis finds no evidence of a strong interaction between stress and 5-HTTLPR genotype contributing to the development of depression. *Mol Psychiatry* 2018; **23:** 133.

7 Beitchman JH, Baldassarra L, Mik H, et al. Serotonin transporter polymorphisms and persistent, pervasive childhood aggression. *Am J Psychiatry* 2006; **163:** 1103–1105.

8 Ficks CA, Waldman ID. Candidate genes for aggression and antisocial behavior: a meta-analysis of association studies of the 5HTTLPR and MAOA-uVNTR. *Behav Genet* 2014; **44:** 427–444.

9 Hessl D, Tassone F, Cordeiro L, et al. Brief report: Aggression and stereotypic behavior in males with fragile X syndrome—Moderating secondary genes in a "single gene" disorder. *J Autism Dev Disord* 2008; **38:** 184–189.

10 Brune CW, Kim S-J, Salt D, Leventhal BL, Lord C, Cook Jr M, EH. 5-HTTLPR genotype-specific phenotype in children and adolescents with autism. *Am J Psychiatry* 2006; **163:** 2148–2156.

11 May ME, Lightfoot DA, Srour A, Kowalchuk RK, Kennedy CH. Association between serotonin transporter polymorphisms and problem behavior in adult males with intellectual disabilities. *Brain Res* 2010; **1357:** 97–103.

12 Manor I, Eisenberg J, Tyano S, et al. Family-based association study of the serotonin transporter promoter region polymorphism (5-HTTLPR) in attention deficit hyperactivity disorder. *Am J Med Genet* 2001; **105:** 91–95.

13 Zoroğlu SS, Erdal ME, Alaşehirli B, et al. Significance of serotonin transporter gene 5-HTTLPR and variable number of tandem repeat polymorphism in attention deficit hyperactivity disorder. *Neuropsychobiology* 2002; **45:** 176–181.

14 Gadow KD, DeVincent CJ, Siegal VI, et al. Allele-specific associations of 5-HTTLPR/rs25531 with ADHD and autism spectrum disorder. *Prog Neuropsychopharmacol Biol Psychiatry* 2013; **40:** 292–297.

15 Caspi A, Sugden K, Moffitt TE, et al. Influence of life stress on depression: moderation by a polymorphism in the 5-HTT gene. *Science* 2003; **301:** 386–389.

16 Karg K, Burmeister M, Shedden K, Sen S. The serotonin transporter promoter variant (5-HTTLPR), stress, and depression meta-analysis revisited: evidence of genetic moderation. *Arch Gen Psychiatry* 2011; **68**(5)**:** 444-454.

17 Haenisch B, Herms S, Mattheisen M, Steffens M, Breuer R, Strohmaier J *et al.* Genome-wide association data provide further support for an association between 5-HTTLPR and major depressive disorder. *J Affect Disord* 2013; **146:** 438-440.

18 Serretti A, Cusin C, Lattuada E, Di Bella D, Catalano M, Smeraldi E. Serotonin transporter gene (5-HTTLPR) is not associated with depressive symptomatology in mood disorders. *Mol Psychiatry* 1999; **4:** 280.

19 Shih J, Chen K. MAO-A and-B gene knock-out mice exhibit distinctly different behavior. *Neurobiology* 1999; **7:** 235–246.

20 Sabol SZ, Hu S, Hamer D. A functional polymorphism in the monoamine oxidase A gene promoter. *Hum Genet* 1998; **103:** 273–279.

21 Caspi A, McClay J, Moffitt TE, et al. Role of genotype in the cycle of violence in maltreated children. *Science* 2002; **297:** 851–854.

22 McDermott R, Tingley D, Cowden J, Frazzetto G, Johnson DD. Monoamine oxidase A gene (MAOA) predicts behavioral aggression following provocation. *Proc Natl Acad Sci U S A* 2009; **106:** 2118–2123.

23 Tassone F, Qi L, Zhang W, Hansen RL, Pessah IN, Hertz-Picciotto I. MAOA, DBH, and SLC6A4 variants in CHARGE: a case–control study of autism spectrum disorders. *Autism Res* 2011; **4:** 250–261.

24 Cohen I, Liu X, Schutz C, et al. Association of autism severity with a monoamine oxidase A functional polymorphism. *Clin Genet* 2003; **64:** 190–197.

25 Syagailo YV, Stöber G, Gräßle M, et al. Association analysis of the functional monoamine oxidase A gene promoter polymorphism in psychiatric disorders. *Am J Med Genet* 2001; **105:** 168–171.

26 Kunugi H, Ishida S, Kato T, et al. A functional polymorphism in the promoter region of monoamine oxidase-A gene and mood disorders. *Mol Psychiatry* 1999; **4:** 393.

27 Gutiérrez B, Arias B, Gastó C, et al. Association analysis between a functional polymorphism in the monoamine oxidase A gene promoter and severe mood disorders. *Psychiatr Genet* 2004; **14:** 203–208.

28 Eisenberg J, Mei-Tal G, Steinberg A, et al. Haplotype relative risk study of catechol-O-methyltransferase (COMT) and attention deficit hyperactivity disorder (ADHD): Association of the high-enzyme activity val allele with adhd impulsive-hyperactive phenotype. *Am J Med Genet* 1999; **88:** 497–502.

29 DeYoung CG, Getchell M, Koposov RA, et al. Variation in the catechol-O-methyltransferase Val158Met polymorphism associated with conduct disorder and ADHD symptoms among adolescent male delinquents. *Psychiatr Genet* 2010; **20:** 20.

30 Gothelf D, Michaelovsky E, Frisch A, et al. Association of the low-activity COMT 158 Met allele with ADHD and OCD in subjects with velocardiofacial syndrome. *Int J Neuropsychopharmacol* 2007; **10:** 301–308.

31 Nikolac PM, Nedic EG, Stefulj J, et al. Association between the polymorphisms of the selected genes encoding dopaminergic system with ADHD and autism. *Psychiatry Res* 2013; **215**: 260–261.

32 Biederman J, Kim JW, Doyle AE, et al. Sexually dimorphic effects of four genes (COMT, SLC6A2, MAOA, SLC6A4) in genetic associations of ADHD: a preliminary study. *Am J Med Genet B: Neuropsychiatr Genet* 2008; **147:** 1511–1518.

33 Qian Q, Wang Y, Zhou R, et al. Family-based and case-control association studies of catechol-O-methyltransferase in attention deficit hyperactivity disorder suggest genetic sexual dimorphism. *Am J Med Genet B: Neuropsychiatr Genet* 2003; **118:** 103–109.

34 Kotler M, Barak P, Cohen H, et al. Homicidal behavior in schizophrenia associated with a genetic polymorphism determining low catechol O-methyltransferase (COMT) activity. *Am J Med Genet* 1999; **88:** 628–633.

35 Strous RD, Nolan KA, Lapidus R, Diaz L, Saito T, Lachman HM. Aggressive behavior in schizophrenia is associated with the low enzyme activity COMT

polymorphism: a replication study. *Am J Med Genet B: Neuropsychiatr Genet* 2003; **120:** 29–34.

36 Tosato S, Bonetto C, Di Forti M, et al. Effect of COMT genotype on aggressive behaviour in a community cohort of schizophrenic patients. *Neurosci Lett* 2011; **495:** 17–21.

37 Lewis M, Kim S-J. The pathophysiology of restricted repetitive behavior. *J Neurodev Disord* 2009; **1:** 114.

38 Nijmeijer JS, Hartman CA, Rommelse NN, et al. Perinatal risk factors interacting with catechol O-methyltransferase and the serotonin transporter gene predict ASD symptoms in children with ADHD. *J Child Psychol Psychiatry* 2010; **51:** 1242–1250.

39 Pooley E, Fineberg N, Harrison P. The met 158 allele of catechol-O-methyltransferase (COMT) is associated with obsessive-compulsive disorder in men: case–control study and meta-analysis. *Mol Psychiatry* 2007; **12:** 556.

40 James SJ, Melnyk S, Jernigan S, et al. Metabolic endophenotype and related genotypes are associated with oxidative stress in children with autism. *Am J Med Genet B: Neuropsychiatr Genet* 2006; **141:** 947–956.

41 Ohara K, Nagai M, Suzuki Y, Ohara K. Low activity allele of catechol-o-methyltransferase gene and Japanese unipolar depression. *Neuroreport* 1998; **9:** 1305–1308.

42 Åberg E, Fandiño-Losada A, Sjöholm LK, Forsell Y, Lavebratt C. The functional Val158Met polymorphism in catechol-O-methyltransferase (COMT) is associated with depression and motivation in men from a Swedish population-based study. *J Affect Disord* 2011; **129:** 158–166.

43 Kia-Keating BM, Glatt SJ, Tsuang MT. Meta-analyses suggest association between COMT, but not HTR1B, alleles, and suicidal behavior. *Am J Med Genet B: Neuropsychiatr Genet* 2007; **144:** 1048–1053.
