# Supplementary material for: Genetic modifiers in rare disorders: the case of fragile X syndrome
Source: Eur J Hum Genet. 2020 Aug 29;29(1):173–83. doi: 10.1038/s41431-020-00711-x (PMC7852869; doi:10.1038/s41431-020-00711-x)
Supplement: Supplementary file 2 — Supplementary Information File 2 [file 41431_2020_711_MOESM2_ESM.pdf]

Genetic modifiers in rare disorders: The case of fragile X syndrome

H. Crawford, G. Scerif, L. Wilde, A. Beggs, J. Stockton, P. Sandhu, L. Shelley, C. Oliver & J. P. McCleery

Supplementary Information File 2 Additional information on questionnaire measures used in the study

Demographic Questionnaire

A demographic questionnaire was used to collect participants' background information including their age, gender, mobility, verbal ability (more than 30 words/signs), and information on their diagnosis including who made the diagnosis and when.

The Wessex Scale

The Wessex scale[1] is an informant questionnaire designed to assess social and physical capabilities in children and adults with intellectual disabilities across five subscales (self-help, continence, mobility, speech, and literacy). The Wessex scale has been described as an effective tool for large scale questionnaire studies and has good inter-rater reliability at subscale level for both children and adults with intellectual disabilities.[2] For the purposes of this study, and in line with previous literature,[3–7] the self-help subscale was used to characterise participants' level of ability.

The Challenging Behaviour Questionnaire

The Challenging Behaviour Questionnaire (CBQ)[8] is a seven-item informant questionnaire designed to identify the phenomenology of challenging behavior in individuals with intellectual disabilities. The measure assesses the presence of self-injury, physical aggression, destruction of property and stereotyped behaviour over the last month. Where self-injurious behavior is reported, the severity and presence or absence of eight different topographies are measured. The CBQ demonstrates good inter-rater reliability with coefficients ranging from .61 to .89[8].

The Social Communication Questionnaire

The Social Communication Questionnaire (SCQ)[9] is a 40 item informant questionnaire that assesses characteristics associated with ASD. There are three subscales: social interaction, communication and stereotyped and repetitive behaviour. The SCQ's internal consistency is good ($\alpha = .90$). The SCQ also shows good concurrent validity with both the Autism Diagnostic Interview[10] and the Autism Diagnostic Observation Schedule.[11,12]

The Activity Questionnaire

The Activity Questionnaire (TAQ)[13,14] is an 18 item informant questionnaire designed to assess hyperactivity and impulsivity in individuals with intellectual disabilities. There are three subscales: overactivity, impulsivity and impulsive speech. Because immobile participants are only able to score on four of the six items on the impulsivity subscale, scores are prorated. Internal consistency and test-retest reliability is comparable to other standardised measures of activity.[14]

The Repetitive Behaviour Questionnaire

The Repetitive Behaviour Questionnaire (RBQ)[3] is a 19 item informant questionnaire that is used to assess the presence of repetitive behaviours in individuals with intellectual disabilities. There

are five subscales: stereotyped behaviour, compulsive behaviour, repetitive speech, insistence on sameness and restricted preferences. Robust inter-rater reliability, test-retest reliability, concurrent validity, content validity and internal consistency are reported.[3] Convergent validity between the RBQ and the Repetitive Behaviour subscale of the Autism Screening Questionnaire[15] is good.

The Mood, Interest and Pleasure Questionnaire – Short Form
The Mood, Interest and Pleasure Questionnaire (MIPQ)[16] is a 25-item informant questionnaire designed to assess mood and anhedonia in individuals with intellectual disabilities. There are two subscales: Mood, and Interest and Pleasure. A shortened version of the MIPQ (MIPQ-S),[17] which includes 12 items from the original measure, was used in the current study. The MIPQ-S shows good internal consistency at subscale level and total score (all Cronbach's alpha coefficients $\geq$.79). Test-retest and inter-rater reliability are also good (.97 and .85, respectively).

References

1 Kushlick A, Blunden R, Cox C. A method of rating behaviour characteristics for use in large scale surveys of mental handicap. *Psychol Med* 1973; **3:** 466–478.

2 Palmer J, Jenkins J. The 'Wessex' behaviour rating system for mentally handicapped people: reliability study. *Int J Dev Disabil* 1982; **28:** 88–96.

3 Moss J, Oliver C, Arron K, Burbidge C, Berg K. The prevalence and phenomenology of repetitive behavior in genetic syndromes. *J Autism Dev Disord* 2009; **39:** 572–588.

4 Oliver C, Berg K, Moss J, Arron K, Burbidge C. Delineation of behavioral phenotypes in genetic syndromes: Characteristics of autism spectrum disorder, affect and hyperactivity. *J Autism Dev Disord* 2011; **41:** 1019-1032.

5 Richards C, Moss J, Nelson L, Oliver C. Persistence of self-injurious behaviour in autism spectrum disorder over 3 years: A prospective cohort study of risk markers. *J Neurodev Disord* 2016; **8**.

6 Waite J, Moss J, Beck SR, et al. Repetitive behavior in Rubinstein-Taybi syndrome: Parallels with autism spectrum phenomenology. *J Autism Dev Disord* 2015; **45:** 1238–1253.

7 Wilde L, Eden K, de Vries P, Moss J, Welham A, Oliver C. Self-injury and aggression in adults with tuberous sclerosis complex: Frequency, associated person characteristics, and implications for assessment. *Res Dev Disabil* 2017; **64:** 119–130.

8 Hyman P, Oliver C, Hall S. Self-injurious behaviour, self-restraint, and compulsive behaviours in Cornelia de Lange syndrome. *Am J Intellect Dev Disabil* 2002; **107:**146–154.

9 Rutter M, Bailey A, Lord C. *The Social Communication Questionnaire*. Western Psychological Services: Los Angeles, CA, 2003.

10 Lord C, Rutter M, Le Couteur A. Autism Diagnostic Interview-Revised: A revised version of a diagnostic interview for caregivers of individuals with possible pervasive developmental disorders. *J Autism Dev Disord* 1994; **24:** 659–685.

11 Lord C, Rutter M, DiLavore P, Risi S. *Autism Diagnostic Observation Schedule: Manual*. Western Psychological Services: Los Angeles, CA, 2002.

12 Howlin P, Karpf J. Using the social communication questionnaire to identify "autistic spectrum" disorders associated with other genetic conditions: Findings from a study of individuals with Cohen syndrome. *Autism* 2004; **8:** 175–182.

13 Burbidge C, Oliver C. Activity Questionnaire: Manual for administration and scorer interpretation. *University of Birmingham* 2008.

14 Burbidge C, Oliver C, Moss J, et al. The association between repetitive behaviours, impulsivity and hyperactivity in people with intellectual disability. *J Intellect Disabil Res* 2010; **54:** 1078–1092.

15 Berument SK, Rutter M, Lord C, Pickles A, Bailey A. Autism screening questionnaire: Diagnostic validity. *BJPsych* 1999; **175:** 444–451.

16 Ross E, Oliver C. Preliminary analysis of the psychometric properties of the Mood, Interest & Pleasure Questionnaire (MIPQ) for adults with severe and profound learning disabilities. *Br J Clin Psychol* 2003; **42:** 81–93.

17 Arron K, Oliver C, Moss J, Berg K, Burbidge C. The prevalence and phenomenology of self-injurious and aggressive behaviour in genetic syndromes. *J Intellect Disabil Res* 2011; **55:** 109–120.
